# Bone Turnover in Patients with Chronic Kidney Disease Stage 5D and Healthy Controls — a Quantitative [^18^F]Fluoride PET Study

**DOI:** 10.1007/s11307-023-01834-5

**Published:** 2023-07-11

**Authors:** Dan Fuglø, Anders Løve Paaske Drachmann, Kim Minh Michael Heltø, Lisbeth Marner, Ditte Hansen

**Affiliations:** 1 Department of Nuclear Medicine, Copenhagen University Hospital-Herlev and Gentofte, Copenhagen, Denmark; 2 Department of Anaesthesiology, Copenhagen University Hospital-Herlev and Gentofte, Copenhagen, Denmark; 3 Department of Clinical Physiology and Nuclear Medicine, Copenhagen University Hospital-Bispebjerg and Frederiksberg, Copenhagen, Denmark; 4 Department of Nephrology, Copenhagen University Hospital-Herlev and Gentofte, Copenhagen, Denmark; 5 Department of Clinical Medicine, University of Copenhagen, Copenhagen, Denmark

**Keywords:** Renal osteodystrophy, Gjedde-Patlak plot, Positron emission tomography

## Abstract

**Background:**

Chronic kidney disease (CKD) is prevalent in the aging population and increases the risk of fracture 2–4 times. We compared optimized quantitative [^18^F]fluoride PET/CT methods to the reference standard with arterial input function (AIF) to identify a clinically accessible method for evaluation of bone turnover in patients with CKD.

**Methods:**

Ten patients on chronic hemodialysis treatment and ten control patients were recruited. A dynamic 60-min [^18^F]fluoride PET scan was obtained from the 5th lumbar vertebra to the proximal femur simultaneously with arterial blood sampling to achieve an AIF. Individual AIFs were time-shifted to compute a population curve (PDIF). Bone and vascular volumes-of-interest (VOIs) were drawn, and an image-derived-input-function (IDIF) was extracted. PDIF and IDIF were scaled to plasma. Bone turnover (*K*_*i*_) was calculated with the AIF, PDIF, and IDIF and bone VOIs using a Gjedde-Patlak plot. Input methods were compared using correlations and precision errors.

**Results:**

The calculated *K*_*i*_ from the five non-invasive methods all correlated to the *K*_*i*_ from the AIF method with the PDIF scaled to a single late plasma sample showing the highest correlations (*r* > 0.94), and the lowest precision error of 3–5%. Furthermore, the femoral bone VOI’s correlated positively to p-PTH and showed significant differences between patients and controls.

**Conclusions:**

Dynamic 30 min [^18^F]fluoride PET/CT with a population based input curve scaled to a single venous plasma sample is a feasible and precise non-invasive diagnostic method for the assessment of bone turnover in patients with CKD. The method may potentially allow for earlier and more precise diagnosis and may be useful for assessment of treatment effects, which is crucial for development of future treatment strategies.

**Supplementary Information:**

The online version contains supplementary material available at 10.1007/s11307-023-01834-5.

## Background

Chronic kidney disease (CKD) is present in almost 10% of the adult population [1]. As kidney function declines, the risk of fracture increases and at the time of end stage kidney disease, the risk of fracture is 2–4 times elevated [2]. In addition, fractures in patients with chronic kidney disease are associated with increased morbidity and mortality [3]. The bone disease present in patients with CKD is renal osteodystrophy, in which bone turnover may be highly varying from low bone turnover, where no bone is resorbed or formed, to high bone turnover, where a high level of bone resorption and formation occurs. Both low and high bone turnover may lead to pathological bone changes and increased fracture risk [4]. Knowledge of the present bone turnover is of great clinical importance, as bisphosphonates reduce bone turnover and is considered optimal for the high turnover bone disease, whereas teriparatide increases bone turnover and is considered best treatment of the low bone turnover disease [5].

Presently, the gold standard for assessment of bone turnover is histomorphometric examination of an invasive bone biopsy, which is seldom performed leaving many patients. Changes over time in bone turnover cannot be obtained by a single biopsy precluding assessment of treatment effects unless sequential biopsies are performed. Furthermore, the biopsy site is of importance as local changes may bias or induce measurement errors in the results that are based on a single sample. Biochemical assessment of bone turnover by circulating bone turnover markers has been explored but adds only sparsely to the accuracy found by the routinely measurement of parathyroid hormone [6].

[^18^F]Fluoride is a widely available bone seeking tracer that allows for quantitative assessment of bone metabolism using positron emission tomography (PET). The accumulation of [^18^F]fluoride in bone is dependent on perfusion and more importantly bone remodeling, i.e., osteoblast activity, as it is built irreversibly into fluorapatite. [^18^F]Fluoride PET/CT appears as a promising method to assess the bone turnover in patients with CKD [7, 8], as compared to invasive histomorphometric examinations in 26 and 7 patients, respectively. The reference standard for assessment of quantitative bone metabolism using [^18^F]fluoride PET/CT requires long dynamic imaging simultaneously with arterial blood samples, which is a strenuous method for the patient. A number of studies have tried to obviate the need for arterial cannulation but only few of the studies have compared to the reference standard with arterial cannulation. Blake et al. [9] proposed a semipopulation-based input function (SPIF) for [^18^F]fluoride PET/CT studies based on nine healthy women with full arterial plasma sampling [10]. The SPIF is a combination of a population-based curve scaled to injected activity combined with venous plasma samples from 30 to 60 min after tracer injection fitted to an exponential function, the latter accounting for around 75% of the total area-under-the-curve (AUC). The SPIF method has been implemented by Frost et al. [7] with PET scans of lumbar spine and a whole body scan in 7 patients with suspected low bone turnover and 12 with osteoporosis. Interestingly, they found no difference in the bone turnover marker, *K*_*i*_, between the subjects with low bone turnover and those without and between those with renal failure and those with osteoporosis. Aaltonen et al. [11] used an image-derived input function (IDIF) from the abdominal aorta in the scanner field of view, and Messa et al. [12] used heated venous blood sampling. Aaltonen et al. compared the fractional uptake rate (FUR), which is an approximation of *K*_*i*_, to the histomorphometric examination of a bone biopsy in 26 dialysis patients and showed a clear correlation with a sensitivity and specificity of 76 and 78% for detecting low bone turnover [11].

Vrist et al. [13] examined 17 patients with CKD using the left ventricle of the heart as an IDIF and thoracic spine with a considerable correction for partial volume effects. The IDIF was subsequently used to construct their own SPIF to allow for future static whole body imaging. The caveat of the SPIF method is the need for several venous blood samples during the scan, which may be difficult and laborious to obtain, and further simplifications using static imaging have been proposed by, e.g., Siddique et al. [14]. Thus, a number of studies have proposed methods for non-invasive image-based quantitative assessment of bone turnover, although only very few studies have compared to the reference standard using arterial cannulation.

The aim of the present study was to optimize the [^18^F]fluoride PET/CT method as an accessible method in the everyday clinic for evaluation of bone turnover in patients with CKD. Different input functions were examined, and the influence of the presence of end stage kidney disease or normal kidney function was explored.

## Methods

### Participants

Ten patients on chronic hemodialysis treatment (> 3 months) (CKD stage 5D) having an arteriovenous fistula as a dialysis access were recruited from the outpatient clinic at Copenhagen University Hospital Herlev, Denmark. Ten control patients were recruited among patients already planned to a [^18^F]fluoride PET to examine for prostate cancer with a PSA < 40μg/L and no signs of bone metastases on the [^18^F]fluoride PET (*n* = 4) and among healthy persons by advertisement at www.foersoegsperson.dk (*n* = 6). The control participants had an estimated glomerular filtration rate (eGFR) by the CKD-EPI formula ≥ 60 mL/min/1.73 m^2^. All participants were adult ≥ 18 years of age and gave written consent after written and oral information, according to ethical principles of the Helsinki Declaration. The study was approved by the Regional Ethics Committee (H-17040409) and the Danish Data Protection Agency (HGH-2018-021, I-Suite nr.: 06257).

### Image Acquisition

PET/CT was performed on Siemens Biograph mCT (Siemens Healthineers, Erlangen, Germany). A dynamic PET scan was obtained from the 5th lumbar vertebra to the intertrochanteric region of the proximal femur and were recorded in list mode for 60 min following automated intravenous injection of 200 MBq [^18^F]fluoride using a Medrad Intego PET Infusion System (Bayer HealthCare, Netherlands). The field of view included bone structures of interest for kidney disease and risk of fracture on the cost of the larger intrathoracic blood reservoirs that would have eased the extraction of an IDIF. Images were reconstructed in time frames of 5 s for the first 2 min in order to capture the vascular bolus passage in the iliac arteries and then in frames of 4 × 30 s, 8 × 1 min, and 12 × 4 min. Spatial reconstruction was performed iteratively (ordered subset expectation maximization, 4 iterations; 21 subsets) with a matrix size of 400 × 400 and a final resolution of 0.8 × 0.8 × 5 mm^3^. A static PET scan was subsequently performed from vertex to knees, and low dose CT was used for attenuation correction and bone-VOI delineation.

### Volumes of Interest

**Fig. 1:**
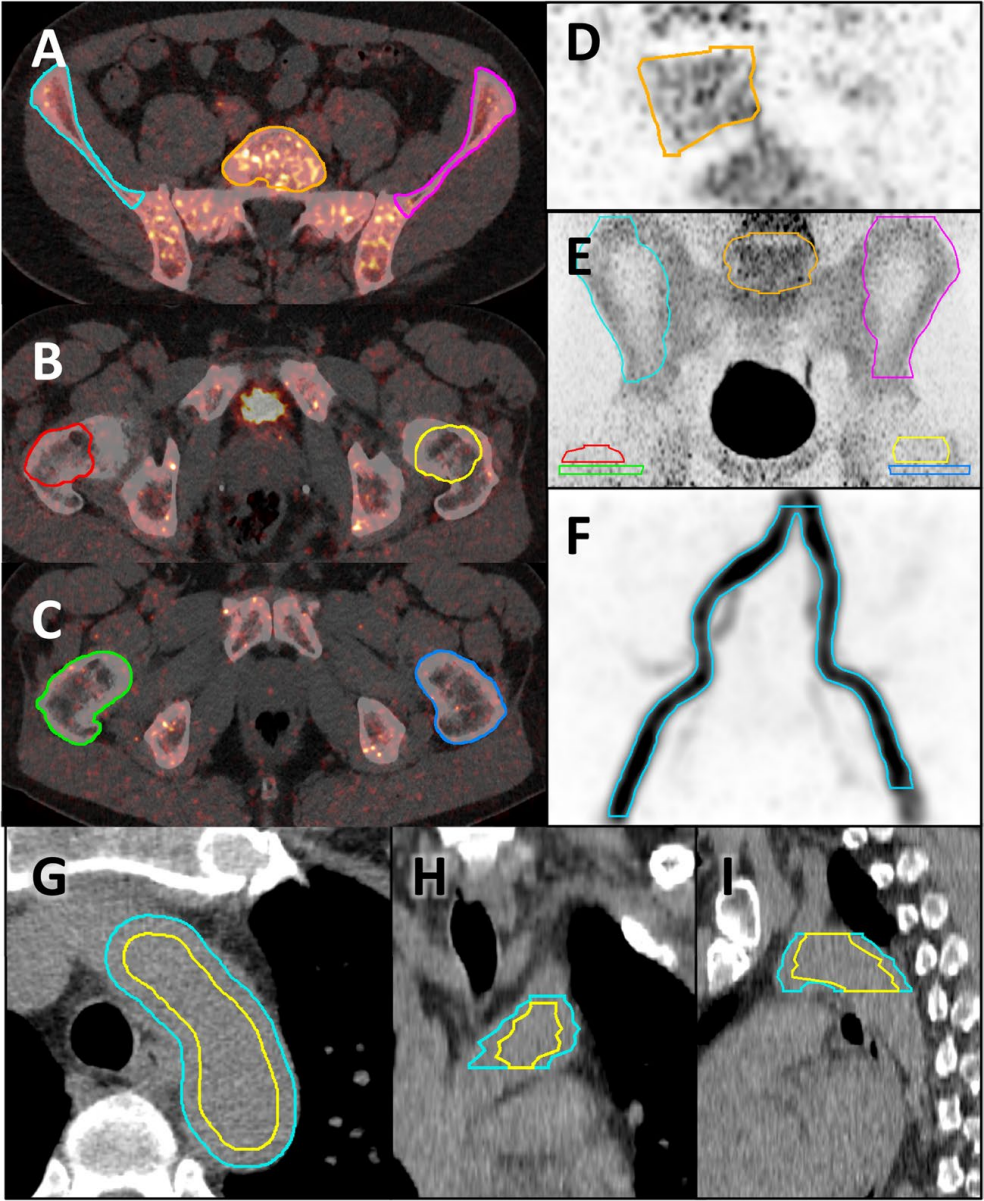
Short title: Volumes of interest. Detailed legend: example of bone VOIs (**A**–**E**) and vascular VOIs in the common and external iliac arteries (**F**) and aortic arch (**G**-**I**)

Volumes of interest (VOIs) were drawn using the contouring tools in MIM Encore (MIM Software, USA). A vascular VOI covering the common iliac arteries and part of the external iliac arteries was drawn by averaging dynamic PET frames of the arterial phase of the bolus passage of the tracer and placing seed points in the common iliac arteries (Fig. 1F). Bone VOIs were drawn on the CT series around the L5, iliac wings, femoral necks, and proximal femoral shafts by manually placing VOIs covering the bones and using edge detection scripts developed by MIM to crop them to the cortical boundary (Fig. 1A–E). In order to test for the effect of inter-observer variation in VOI placement, two experienced nuclear medicine physicians drew VOIs around the left iliac wing, femoral neck, femoral shaft, and the L5 on all 20 subjects and intraclass correlation coefficients (ICC) were calculated using the mean SUV of the last PET frame. Patients with a hip prosthesis were excluded from the analysis of the corresponding hip region. If the proximal femoral shaft was not included in the PET field of view it was excluded from the analysis. A vascular VOI in the aortic arch was drawn on the late static PET series by manually placing a VOI around the aortic arch and cropping the peripheral 5 mm closest to the vessel wall in order to reduce partial volume effects (Fig. 1G–I). Motion correction of the dynamic PET series was performed in PMOD software (PMOD Technologies, Switzerland), and time activity curves (TACs) were formed from the bone VOIs and iliac artery VOIs.

### Arterial Input Functions

Arterial blood samples were collected at time 0 and every 8 to 10 s for the first 2 min followed by samples at 2½, 3, 4, 5, 6, 8, 10, 15, 20, 25, 30, 40, 50, and 60 min. Venous blood samples were collected at 30, 40, 50, and 60 min. The activities in whole blood and plasma were measured on a Hidex Automatic Gamma Counter (Hidex, Turku, Finland) in the 400–1200 keV window, and sample mass weight was determined by automatic weighing of vials in the gamma counter before and after sampling. Individual whole blood/plasma ratios were averaged to generate a population-based value to correct the image-derived input functions (IDIF).

### Population-Derived Input Functions

**Fig. 2:**
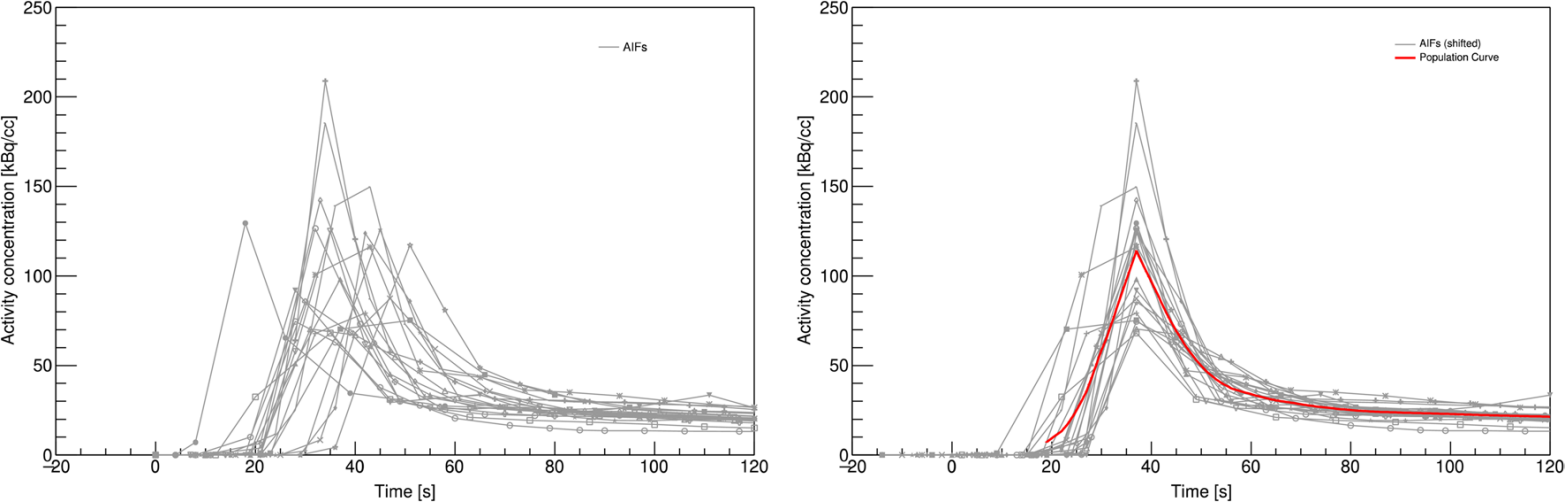
Short title: Individual and population-based AIFs. Detailed legend: arterial plasma input function (AIF) for all the participants shown for the first 2 min after injection. The left plot shows the unscaled and unshifted AIFs. In the right plot, all the AIFs have been time-shifted to mean time-to-peak. The resulting population curve for the total sample of patients is shown in red

All individual AIFs were time-shifted to align the peaks in order to compute the population curves for the CKD group, the control group, and the entire population. Figure 2 shows the unshifted and shifted AIFs for all the participants, with the total population curve (mean curve) shown in red in the latter. The resulting curves were then scaled and time-shifted to generate individual PDIFs.

When generating the individual PDIF, the subject’s AIF was omitted from the population curve to avoid circular analysis. Scaling of the population curves was performed using either plasma activity at time 50–60 min (p-PDIF) or activity in the aortic VOI divided by a whole-blood/plasma ratio of 0.77 (a-PDIF). As the aortic concentration was measured after the end of the dynamic scan, the tail of the PDIFs was fitted using an exponential function and extrapolated to match the time of the aortic value. Time shifting of the PDIF was performed using the peak value of the iliac TAC or a mean peak value.

### Image-Derived Input Functions

The TAC from iliac arteries was used to create three IDIFs for each subject. Using the iliac TAC divided by a whole-blood/plasma ratio of 0.77 (average value for the population, as we found no significant differences between groups), an IDIF was generated (r-IDIF). In order to compensate for partial volume effects, two additional IDIFs were created by scaling r-IDIF to activity in the aortic arch (a-IDIF) and plasma (p-IDIF) in a similar fashion, as described for the PDIF.

### Gjedde-Patlak Analysis

As fluoride is highly irreversible bound with *k*_4_ being low (around 0.01 min^−1^) [9], the irreversible Gjedde-Patlak plot was used to calculate the unidirectional uptake rate constant of [^18^F]fluoride from plasma to bone [*K*_*i*_ (ml min^−1^ ml^−1^)] using PMOD Software (PMOD Technologies LLC, Switzerland). *K*_*i*_ was determined from the slope in the last 30 min of the dynamic scan time (t* fixed to 30 min) and was calculated for all bone VOIs using the various input functions, i.e., AIF, IDIFs, and PDIFs.

### Statistical Analysis

A significance level of 0.05 was adopted throughout. Group comparisons were performed with unpaired *t*-test or Mann Whitney *U*. Pearson’s correlation coefficients were used for assessing correlations.

Precision errors to estimate difference between optimized methods and the reference standards were calculated as $\sqrt{Mean\left({\left({K}_{i_{model}}-{K}_{i_{reference}}\right)}^2\right)}/ Mean\left({K}_{i_{reference}}\right)\bullet 100\%$. Statistical analysis was performed using SPSS Statistics (SPSS Inc., USA).

## Results

Demographics are presented in Table 1. The two groups had the same distribution of sex and were of similar age. The patients had a numeric higher frequency of former fracture than the control group. As expected, the patients differed from the control group in terms of medication and levels of p-ionized calcium, p-phosphate, and p-iPTH. Within the control group, the patients with prostate cancer were older (median 75.5 years; range 75–81) than the healthy controls (median 57.0 years; range 52-66) (*p* = 0.019). There were no other significant differences between the patients with prostate cancer and the healthy controls within the control group.

**Table 1:** Demographics

	Patients (*n* = 10)	Controls (*n* = 10)
Sex (male (%))	8 (80%)	9 (90%)
Age (years)	71 (64–75)	69 (55–75)
eGFR (mL/min/1.73 m^2^)	NA	81 (9)
Duration of dialysis (month)	32 (19–36)	NA
Previous fracture	6 (60%)	2 (20%)
Vitamin D therapy (active/native)	7 (70%)/3 (30%)	0
Phosphate binders (calcium/non-calcium containing)	5 (50%)/6 (60%)	0
Calcimimetic therapy	3 (30 %)	0
p-ionized calcium (mmol/L)	1.17 (0.08)*	1.26 (0.05)
p-phosphate (mmol/L)	1.73 (0.68)*	1.07 (0.21)
p-magnesium (mmol/L)	0.93 (0.18)	0.83 (0.04)
p-iPTH (pmol/L)	47 (22–57)*	6 (5–8)
p-alkaline phosphatase (U/L)	114 (62–137)	75 (60–87)

Mean (SD) or median (interquartile range) or numbers (percent)

* *p* < 0.05 compared to control group unpaired *t*-test or Mann Whitney *U*

**Fig. 3:**
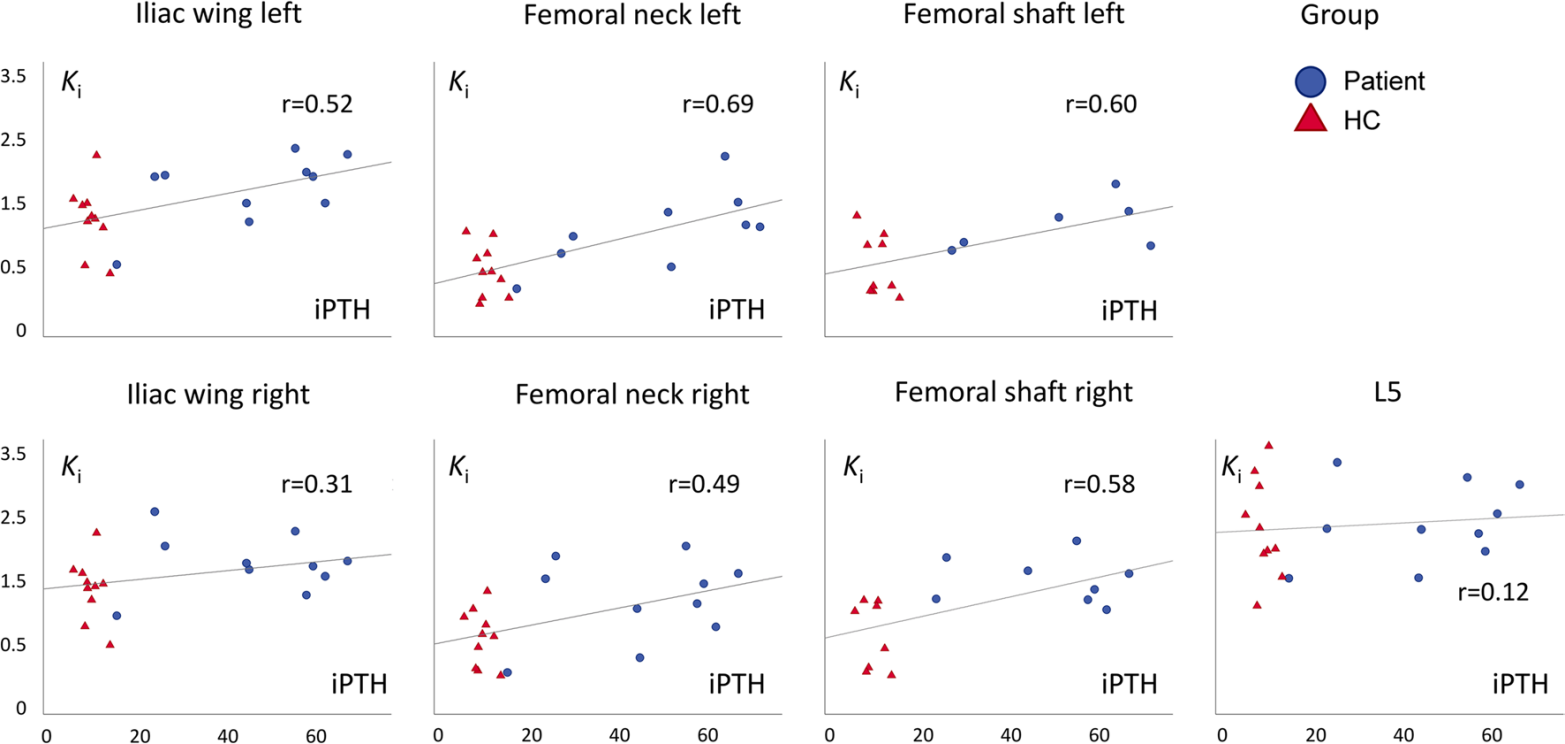
Short title: *K*_*i*_ and iPTH. Detailed legend: scatter plots of *K*_*i*_ (mL/(min × 100 mL) as a function of iPTH (pmol/L) for patients and healthy controls. *K*_*i*_ is calculated for each VOI using the arterial plasma input function

The first 2 min of the measured arterial input curves for all subjects with and without time shift is shown in Fig. 2. Note the red curve as the resulting population curve (PDIF), which was based on all participants, as no differences between the curves for patients and controls were found, and the differences between the curves of the prostate cancer and healthy control subgroups were negligible. Furthermore, shifting the PDIF to the individual peak did not change the resulting *K*_*i*_ values. Venous plasma samples in the first subjects examined were contaminated with the injected activity, as they were drawn in the cannula used for [^18^F]fluoride injection. Subsequently, subjects had an additional venous cannula, and these samples were comparable to the plasma values of the arterial samples. The effect of inter-observer variation in VOI placement was very low shown by ICC values between 0.97 and 0.99 for the different VOIs. Please refer to Table 2 for calculated *K*_*i*_ for the seven chosen bone regions with the top row (AIF) being the reference standard. Please note the higher bone turnover in patients, as compared to controls in femoral VOIs and a tendency in the iliac crest, while no difference was found in the vertebra. Table 3 shows the correlation coefficients and the precision errors between the non-invasive methods, compared to the reference standard. Note that all correlation coefficients are above 0.94 for the PDIF scaled to a single plasma sample, which also show the lowest precision error of 2.7–5.4%. Supplemental Figure 1 shows Bland–Altman plots of the five non-invasive methods, compared to AIF. Again, note the very small deviations between the *K*_*i*_ values using p-PDIF. A positive correlation between PTH and *K*_*i*_ were found in the left iliac wing and the femoral regions (Fig. 3).

**Table 2:** *K*_*i*_ values

	Iliac wing		Femoral neck		Femoral body		L5
	Left	Right	Left	Right	Left	Right	
	Mean *K*_*i*_ (SD)	Mean *K*_*i*_ (SD)	Mean *K*_*i*_ (SD)	Mean *K*_*i*_ (SD)	Mean *K*_*i*_ (SD)	Mean *K*_*i*_ (SD)	Mean *K*_*i*_ (SD)
AIF							
All	1.88 (0.50)	1.95 (0.44)	1.21 (0.52)	1.35 (0.54)	1.24 (0.47)	1.51 (0.55)	2.66 (0.60)
Patients	2.08 (0.47)	2.13 (0.40)	1.52 (0.52)*	1.61 (0.56)*	1.58 (0.33)*	1.90 (0.31)**	2.70 (0.54)
Controls	1.67 (0.45)	1.77 (0.41)	0.94 (0.35)	1.09 (0.38)	1.01 (0.41)	1.12 (0.45)	2.63 (0.66)
p-IDIF							
All	1.73 (0.57)	1.81 (0.55)	1.17 (0.58)	1.23 (0.56)	1.13 (0.54)	1.40 (0.59)	2.44 (0.64)
Patients	2.03 (0.49)*	2.11 (0.45)*	1.56 (0.52)**	1.53 (0.55)*	1.53 (0.36)*	1.81 (0.38)**	2.65 (0.51)
Controls	1.43 (0.49)	1.51 (0.46)	0.82 (0.37)	0.94 (0.39)	0.87 (0.47)	0.99 (0.47)	2.23 (0.68)
a-IDIF							
All	1.52 (0.49)	1.58 (0.46)	1.01 (0.47)	1.08 (0.47)	0.96 (0.43)	1.22 (0.48)	2.15 (0.57)
Patients	1.76 (0.41)*	1.82 (0.37)*	1.31 (0.41)**	1.31 (0.46)	1.25 (0.25)*	1.55 (0.24)**	2.30 (0.48)
Controls	1.28 (0.45)	1.35 (0.42)	0.74 (0.34)	0.85 (0.36)	0.77 (0.42)	0.88 (0.41)	1.99 (0.61)
r-IDIF							
All	1.86 (0.55)	1.97 (0.56)	1.20 (0.37)	1.31 (0.52)	1.22 (0.37)	1.43 (0.46)	2.67 (0.75)
Patients	1.77 (0.65)	1.84 (0.68)	1.33 (0.41)	1.35 (0.65)	1.32 (0.37)	1.56 (0.46)	2.27 (0.72)
Controls	1.96 (0.41)	2.09 (0.38)	1.09 (0.28)	1.28 (0.34)	1.15 (0.36)	1.31 (0.42)	3.07 (0.54)
p-PDIF							
All	1.88 (0.54)	1.96 (0.46)	1.22 (0.53)	1.35 (0.56)	1.23 (0.48)	1.50 (0.55)	2.66 (0.62)
Patients	2.12 (0.52)	2.15 (0.42)	1.54 (0.54)*	1.62 (0.58)*	1.58 (0.33)*	1.89 (0.32)**	2.72 (0.57)
Controls	1.65 (0.45)	1.76 (0.41)	0.94 (0.34)	1.09 (0.37)	0.99 (0.40)	1.10 (0.44)	2.61 (0.67)
a-PDIF							
All	1.66 (0.48)	1.73 (0.41)	1.07 (0.42)	1.18 (0.45)	1.06 (0.37)	1.29 (0.42)	2.36 (0.62)
Patients	1.69 (0.48)	1.72 (0.39)	1.21 (0.44)	1.29 (0.49)	1.21 (0.28)	1.50 (0.27)*	2.16 (0.48)
Controls	1.63 (0.48)	1.74 (0.44)	0.94 (0.36)	1.08 (0.39)	0.96 (0.39)	1.07 (0.42)	2.58 (0.68)

Mean *K*_*i*_ values (mL/(min × 100 mL)) from Patlak analysis of bone VOIs using arterial input functions from arterial plasma and fitted image and population based curves. Statistical significance between patient and healthy control groups using independent sample *t*-test is denoted by * (*p* < 0.05) or ** (*p* < 0.01)

*a-IDIF* Image-derived input function (IDIF) scaled using activity in the aortic arch, *r-IDIF* IDIF divided by whole-blood plasma ratio, *p-PDIF* population-derived input function (PDIF) scaled using a late arterial plasma concentration, *a-PDIF* PDIF scaled using activity in the aortic arch

**Table 3:** Correlation coefficients and precision errors between *K*_*i*_ AIF, and *K*_*i*_ model

	Iliac wing		Femoral neck		Femoral body		L5
	Left	Right	Left	Right	Left	Rght	
p-IDIF							
All	0.876**	0.865**	0.976**	0.910**	0.980**	0.949**	0.845**
Patients	0.773**	0.783**	0.980**	0.847**	0.964**	0.768*	0.837**
Controls	0.932**	0.897**	0.958**	0.955**	0.970**	0.975**	0.903**
Precision error	16.7%	15.9%	11.4%	19.3%	11.1%	12.8%	15.5%
a-IDIF							
All	0.888**	0.866**	0.958**	0.921**	0.975**	0.971**	0.822**
Patients	0.814**	0.802**	0.945**	0.874**	0.979**	0.866**	0.804**
Controls	0.915**	0.872**	0.949**	0.946**	0.972**	0.977**	0.865**
Precision error	22.9%	22.4%	20.5%	25.5%	20.8%	19.3%	23.5%
r-IDIF							
All	0.478*	0.448*	0.810**	0.658**	0.708**	0.602*	0.472*
Patients	0.493	0.579	0.734*	0.663*	0.409	0.315	0.403
Controls	0.842**	0.710*	0.927**	0.880**	0.922**	0.853**	0.833**
Precision error	28.7%	27.6%	24.9%	32.9%	23.6%	27.4%	26.5%
p-PDIF							
All	0.984**	0.977**	0.996**	0.992**	0.997**	0.997**	0.982**
Patients	0.969**	0.948**	0.993**	0.987**	0.988**	0.981**	0.960**
Controls	0.998**	0.998**	0.998**	0.998**	0.999**	0.999**	0.998**
Precision error	5.4%	5.0%	4.1%	5.2%	2.7%	2.8%	4.4%
a-PDIF							
All	0.863**	0.806**	0.935**	0.920**	0.932**	0.931**	0.841**
Patients	0.881**	0.825**	0.954**	0.933**	0.946**	0.838**	0.831**
Controls	0.966**	0.956**	0.978**	0.975**	0.980**	0.980**	0.967**
Precision error	18.0%	17.9%	19.7%	20.2%	17.8%	18.7%	17.1%

Correlation coefficients between *K*_*i*_ values using the reference arterial plasma input function (AIF) and image- and population-derived input functions. Precision errors are denoted in bold and are calculated as $\frac{\sqrt{Mean\left({\left({K}_{i_{model}}-{K}_{i_{reference}}\right)}^2\right)}}{Mean\left({K}_{i_{reference}}\right)}\bullet 100\%$

*a-IDIF* image-derived input function (IDIF) scaled using activity in the aortic arch, *r-IDIF* IDIF divided by whole-blood plasma ratio; *p-PDIF* population-derived input function (PDIF) scaled using a late arterial plasma concentration, *a-PDIF* PDIF scaled using activity in the aortic arch

* denote significant correlation at the 0.05 (*) or 0.01 (**) level

## Discussion

In the aging population, chronic kidney disease is increasingly prevalent, and the resulting increased risk of fracture may lead to significant morbidity and even mortality. An easily accessible, non-invasive imaging method for bone turnover may allow for earlier and more precise diagnosis as well as assessment of treatment effects, which is crucial for development of future treatment strategies.

We present a comparison of five non-invasive methods for quantitative bone imaging, compared to the reference standard with arterial plasma sampling in 10 patients with CKD stage 5D and 10 controls. A population-based input curve scaled to a single plasma sample showed the highest correlations (*r* > 0.94) and most favorable precision error of 3–5%, compared to the reference method. Previous image-derived methods compared to arterial plasma measurements found precision errors of 15–20%, and the population-based method using multiple venous samples showed a precision error of 13% [9]. The highly improved precision error in the present study may be due to most of the between-subject variation in the input curve being the scaling of the tail, while the early population-based peak only constitutes a minor fraction, i.e. 2 min (Fig. 2). Furthermore, the Gjedde-Patlak plot is a graphical method based on linearity late in the scan and less dependent on early kinetic variations [15, 16].

We found significantly higher *K*_*i*_ in the femoral VOIs in CKD patients, compared to controls, which was found to correlate to higher PTH values (Fig. 3). This is in line with the increase in bone turnover that occurs with increasing levels of PTH [17], as PTH stimulates bone turnover. The presence of high bone turnover in several of the participants with CKD is in accordance with the high frequency of high bone turnover found in previous studies of bone biopsies from patients on dialysis [18].

The significant differences in the femoral regions suggest that the femoral regions may be more sensitive than iliac crest and vertebra for risk assessment, especially the lumbar vertebra 5 did not show group differences (Table 2) or correlate to PTH (Fig. 3) in the present material. The lack of predictive value of L5 can most likely be explained by degenerative bone changes in a number of subjects. It may also be influenced by the differential effect of PTH on trabecular and cortical bone, where the femoral bone is composed of more cortical bone than the lumbar trabecular bone [19]. L3 that was used in previous studies [11] was not included in the field of view. In the previous study, the measured *K*_*i*_ values in L3 were 0.039 mL/(min × mL) with a cut-off for “low turnover” of 0.04 mL/(min × mL) that was used for comparison to histomorphormetric findings [11]. In comparison, our data showed lower *K*_*i*_ values (L5: 0.027; Table 2) and we have no explanation for the difference between sites, although correction for plasma-whole blood ratio and partial volume correction may explain part of the difference. Future follow-up studies of the femur may suggest valid cut-offs for CKD patients.

The strengths of the study are the sample size and the rigid arterial plasma samples that constitute the reference standard for kinetic modeling. Furthermore, the inclusion of bone VOIs in the iliac crest and femoral neck allows for future direct comparisons to biopsies and risk fracture, as compared to several previous studies of the vertebra. The method can be applied in all bone areas of the body with scan times from 30–60 min after [^18^F]fluoride injection. Limitations include lack of reliable venous blood samples in a number of patients for scaling. Instead we used arterial samples that has been shown to match after 30 min [9]. We suggest that a single plasma sample is drawn at the end of the scan for activity measurements minding not to re-use the cannula for tracer injection. Although the Gjedde-Patlak analysis is robust, it does not account for possible reversible binding within the scan time and the perfusion component *K*_1_ cannot be assessed. If changes in perfusion or binding strength are expected, other methods may be more appropriate. It must be stressed that the presented population curve has not been validated for other kinetic models. Furthermore, it must be stressed that we have not identified a relevant cut-off for high or low bone turnover. Further studies are necessary to evaluate the clinical usefulness.

Patients with CKD who have a high risk of bone fracture can be identified by low bone mineral density (BMD) at dual energy X-ray examination. The underlying renal osteodystrophy may cause a highly differential pattern in bone turnover, which cannot be determined by dual energy X-ray. When bone turnover is unknown, the patients are often left untreated. [^18^F]fluoride PET/CT is promising as the future examination to determine bone turnover in patients with CKD. This will make it possible to treat more patients with osteoporosis and CKD and thereby prevent the high fracture rate in this population. Furthermore, [^18^F]fluoride PET/CT could make it possible to monitor the treatment effect by non-invasive imaging.

## Conclusions

We propose a dynamic 30-min [^18^F]fluoride PET/CT with a population-based input curve scaled to a single venous plasma sample after the scan as a feasible non-invasive diagnostic method for the assessment of bone turnover in patients with CKD.

### Supplementary Information

ESM 1 (PNG 820 kb)

## Data Availability

The datasets generated and/or analyzed during the current study are not publicly available due to Danish legislation on data protection but are available from the corresponding author after approval from the Danish data protection agency.

## Abbreviations

*IDIF*: image-derived input function
*PDIF*: population-derived input function
*AIF*: arterial input function
*wb*: whole blood
*p-IDIF*: IDIF scaled to late plasma concentration
*a-IDIF*: IDIF scaled to late aorta concentration (image value)
*r-IDIF*: IDIF scaled to averaged plasma/wb ratio
*p-PDIF*: PDIF scaled to late plasma concentration
*a-PDIF*: PDIF scaled to late aorta concentration (image value)
