# Supplementary figures and images for: Bone Turnover in Patients with Chronic Kidney Disease Stage 5D and Healthy Controls — a Quantitative [18F]Fluoride PET Study
Source: Mol Imaging Biol. 2023 Jul 11;25(5):815–23. doi: 10.1007/s11307-023-01834-5 (PMC10598153; doi:10.1007/s11307-023-01834-5)

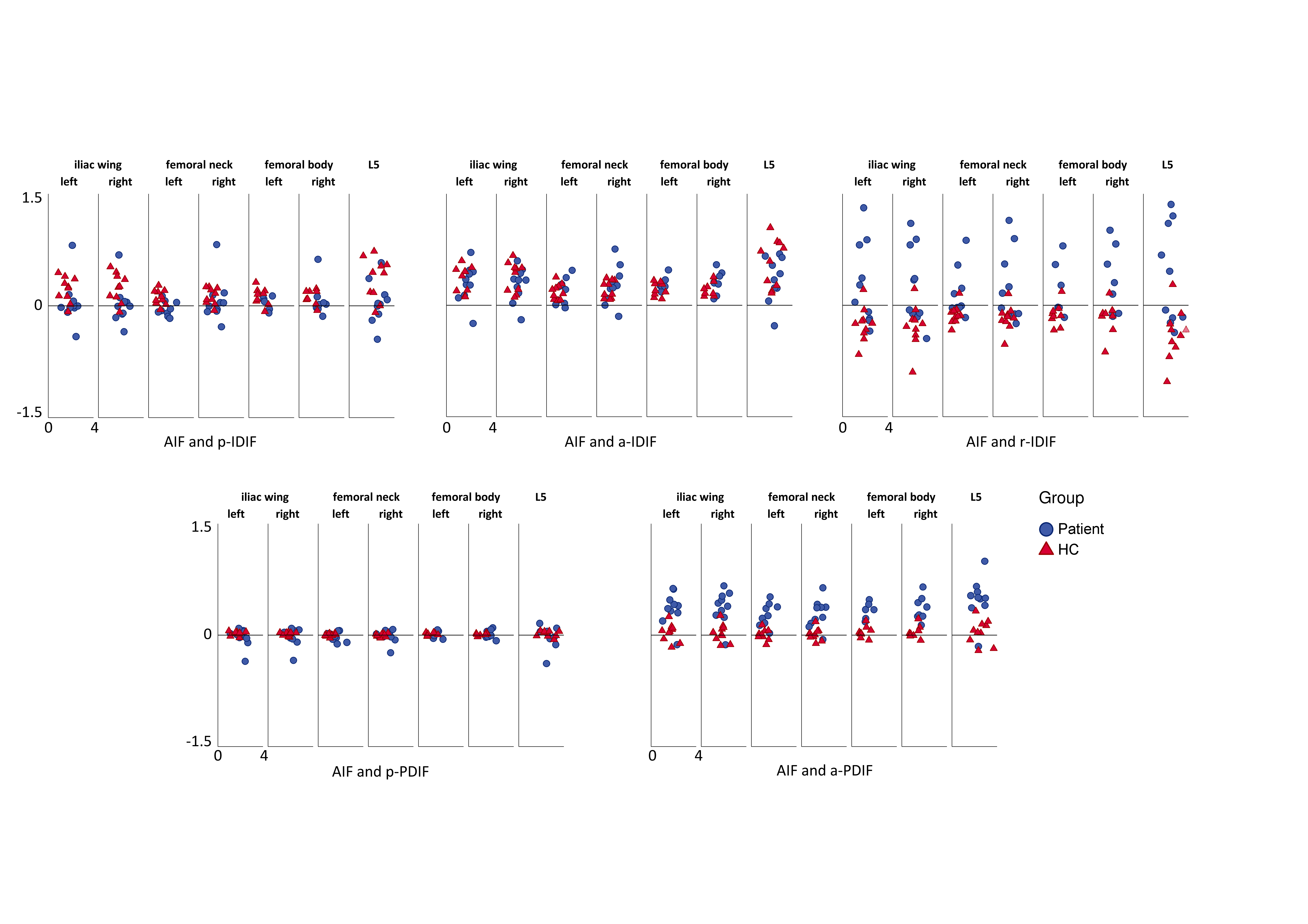

Supplement: Supplementary file 1 — (PNG 820 kb) [file 11307_2023_1834_MOESM1_ESM.png]
